# Association between overweight/obesity and oral health in adults aged 55 and older: a systematic review and meta-analysis

**DOI:** 10.1007/s11357-025-01849-6

**Published:** 2025-09-08

**Authors:** Maryam Raoof, Sami al Chafi, Hamid Reza Tohidinik, Ralph de Vries, Frank Lobbezoo

**Affiliations:** 1 Department of Orofacial Pain and Dysfunction, Academic Centre for Dentistry Amsterdam (ACTA) University of Amsterdam and Vrije Universiteit Amsterdam, Gustav Mahlerlaan 3004, 1081 LA Amsterdam, the Netherlands; 2 Centre for Academic Child Health, Population Health Sciences, Bristol Medical School, University of Bristol, Bristol, UK; 3 Medical Library, Vrije Universiteit, Amsterdam, the Netherlands; 4 Department of Orofacial Pain and Jaw Function, Faculty of Odontology, Malmö University, Malmö, Sweden

**Keywords:** Systematic review, Periodontitis, Dental caries, Tooth wear, Orofacial pain, Obesity, Older adults

## Abstract

The increasing prevalence of overweight/obesity among the elderly has significant implications for oral health due to shared pathophysiological mechanisms. Despite its importance, comprehensive reviews on this topic remain limited. This study investigates the association between overweight/obesity and oral health outcomes in adults aged 55 and older. This systematic review followed PRISMA guidelines and was registered with PROSPERO (CRD42020202108). A systematic literature search was conducted across PubMed, Embase.com, CINAHL, and Web of Science up to November 6, 2023. The study was structured using the Population, Intervention/Exposure, Comparison, Outcome, and Study Design (PICOS) criteria: Population (P) included adults aged ≥ 55 years; Intervention/Exposure (I/E) involved overweight/obesity; Comparison (C) included normal-weight individuals; Outcomes (O) focused on periodontal diseases, dental caries, tooth wear, and orofacial pain; and Study design (S) included case–control, cross-sectional, prospective, and retrospective cohort studies and randomized controlled trials. Eligible studies underwent data extraction and quality assessment using the Newcastle–Ottawa scale. Meta-analyses were conducted using fixed and random effects models in Stata v.18. From 6219 records, 16 studies met the inclusion criteria. Among these, 14 focused on periodontal diseases, while 2 examined dental caries. No studies met the eligibility criteria for tooth wear or orofacial pain. The included studies were predominantly of “good” or “very good” quality. Meta-analysis indicated that overweight or obese older adults had a significantly higher risk of periodontal disease compared with normal-weight counterparts (pooled odds ratio [OR] = 1.49; 95% confidence interval [CI] 1.13–1.97). The association was stronger in studies conducted in Asia (OR = 2.21; 95% CI 1.28–3.81) and in mixed-sex populations (OR = 1.52; 95% CI 1.02–2.28) compared with male-only samples. The two cross-sectional studies on dental caries yielded inconsistent findings, precluding a meta-analysis for this outcome. This systematic review demonstrates a significant association between overweight/obesity and increased prevalence of periodontal disease in adults aged 55 and older. These findings underscore the need to consider oral health as a key component of general health in this population. The current evidence base is insufficient regarding dental caries, tooth wear, and orofacial pain, highlighting the need for high-quality research in these domains.

## Introduction

In 2019, the United Nations reported a global average life expectancy of 72.6 years and projected that by 2050, the population aged 65 and older will double to 1.5 billion, accounting for one in six people globally [92]. Currently, more than 35% of individuals aged 65 and older are obese (over 55% of black women), and if the trend continues, nearly half of the elderly population in the USA will be obese by 2030 [98]. Additionally, the prevalence of overweight is alarmingly high, affecting 78.4% of men and 68.6% of women in this age group [24]. The upward trend in life expectancy has raised concerns about the quality of these additional years, particularly as the prevalence of obesity continues to grow [65].

Body mass index (BMI) is the most commonly used metric to define overweight (BMI ≥ 25) and obesity (BMI ≥ 30) [20, 31, 102]. However, alternative indices, such as waist circumference (WC), waist-to-hip ratio (WHR), and neck circumference (NC), may better predict health risks associated with excess weight [58, 97]. Studies have shown that these metrics correlate strongly with rising BMI and provide valuable insight into the systemic health consequences of overweight/obesity [22]. These conditions are associated with significant societal costs, reduced quality of life, and an increased risk of metabolic, cardiovascular, and inflammatory diseases [22, 36, 70]. Recognizing their shared yet distinct health impacts, this study includes both overweight and obesity within its scope to comprehensively evaluate their relationship with oral health outcomes in older adults.

In older adults, the pathophysiological impact of obesity is more severe due to age-related adipose tissue dysfunction, which exacerbates inflammation, insulin resistance, and metabolic syndrome [44, 82, 91, 93]. Chronic low-grade inflammation in obese older adults has been linked to multimorbidity patterns [1]. Additionally, systemic inflammation has been causally linked to oral health deterioration [33, 90]. The pathophysiological mechanisms of obesity in older adults are complex, involving interactions between aging, hormonal changes (e.g., insulin, leptin, sex hormones, and IGF-1), and vitamin D levels. Immune factors such as pro-inflammatory cytokines, oxidative stress, and mitochondrial dysfunction further contribute, alongside lifestyle factors like diet, physical activity, and smoking, which compound the challenges of obesity in this population [95, 101]. The interplay between obesity and aging can create synergistic effects, such as increased endothelial oxidative stress [91]. Oxidative stress has also been linked to oral health issues [13], with both aging and obesity recognized as significant risk factors for these problems. Khongsirisombat et al. reported that older individuals with overweight or obesity experience increased oral dryness and decreased oral health-related quality of life (OHRQoL) compared to their normal-weight peers [43]. Similarly, a recent large population-based study on older adults linked negative OHRQoL—characterized by discomfort and disability resulting from oral conditions, particularly within the physical pain domain—with a higher likelihood of being obese, overweight, or underweight [16].

Advancements in dentistry have enabled more elderly individuals to retain natural teeth [56] or choose fixed restorations [40, 76]. Maintaining good oral health is essential for overall well-being [5]. Robust evidence clearly indicates that oral health is strongly associated with a spectrum of health outcomes on physical, mental, and social fronts. This connection aligns with the broader concept of holistic health and significantly impacts the quality of life for elderly individuals [52]. Deteriorating oral health in older adults has been associated with increased mortality, frailty, disability, reduced quality of life, hospitalizations, and falls, as well as with reduced quality of [15]. Additionally, elderly individuals with fewer teeth often consume more processed foods, heightening the risk of inadequate nutrition and obesity [37, 46, 66, 68]. Severe periodontal disease further exacerbates health and economic burdens, significantly increasing inpatient and overall medical costs, making it a potential target for reducing healthcare expenses in this population [74]. Understanding the social and economic impact of oral diseases is essential for developing effective interventions, particularly for addressing orofacial health problems in high-risk and vulnerable groups. However, there is a notable lack of systematic reviews or meta-analyses focusing on oral health issues in overweight/obese older adults. A meta-analysis offers the opportunity to synthesize findings from multiple studies, providing a clearer understanding of the issue and identifying knowledge gaps for future research.

The World Health Organization (WHO) and FDI World Dental Federation emphasize that oral health encompasses multiple dimensions, including self-care habits, oral hygiene, function, and mastication ability [29, 67]. In this meta-analysis, we selected periodontal diseases, dental caries, tooth wear, and orofacial pain as indicators of oral health. We hypothesize that these conditions are more prevalent among overweight/obese older adults compared to their normal-weight peers. To investigate this, we employed the Population, Intervention/Exposure, Comparison, Outcome, and Study design (PICOS) criteria, addressing the question: What is the association between overweight/obesity and oral health problems in adults aged 55 and older?

## Methods

### Protocol and registration

This review adheres to the Preferred Reporting Items for Systematic Reviews and Meta-Analyses (PRISMA) guidelines [63]. The protocol was registered with the International Prospective Register of Systematic Reviews (PROSPERO; CRD42020202108). (https:// www.crd.york.ac.uk/prospero/).

### Eligibility criteria

Eligibility was determined using the Population, Intervention/Exposure, Comparison, Outcome, and Study design (PICOS) criteria. Studies considered for inclusion included case–control studies, cross-sectional studies, prospective and retrospective cohort studies, and randomized controlled trials.

The following criteria were applied:

Inclusion criteria:

- Population (P): Older adults (aged ≥ 55 years) of any sex, ethnicity, culture, race, or socio-economic status, from developed or developing countries.
- Intervention/Exposure (I/E): Presence of overweight/obesity, determined by any metric such as body mass index (BMI), waist circumference (WC), waist-to-hip ratio (WHR), or neck circumference (NC).
- Comparison (C): Individuals without overweight/obesity.
- Outcomes (O): Periodontal diseases, dental caries, tooth wear, and orofacial pain.

Exclusion criteria:

- Studies that did not include participants aged ≥ 55 years.
- Articles not published in English.
- Systematic reviews, literature reviews, mini-reviews, short commentaries, letters to the editor, and in vitro studies.

### Search strategy

A comprehensive search of bibliographic databases, including PubMed, Embase.com, CINAHL (Ebsco), and Web of Science, was conducted from their inception to November 6, 2023. The search strategy included keywords and synonyms related to “overweight,” “obesity,” “oral health,” “facial pain,” “aged,” and “elderly.” Terms such as “periodontal diseases,” “dental caries,” “tooth wear,” and “orofacial pain” were categorized under oral health.

References from identified articles were manually reviewed to identify additional relevant studies. Duplicate entries were removed. The detailed search strategies for each database are available in the supplementary material (Sect. 1). Additional searches were conducted in Open Access Theses and Dissertations (OATD) and Open Grey (http://www.opengrey.eu) for relevant theses and grey literature.

### Study selection

Duplicate articles were removed by a medical information specialist using Endnote X20.0.1 (Clarivate™), applying both the Amsterdam Efficient Deduplication (AED) method [61] and the Bramer method [9]. Titles and abstracts (TIAB) were screened using Rayyan (www.rayyan.ai, Qatar Computing Research Institute (QCRI), Doha, Qatar) [62]. Two independent reviewers (M.R. and S.C.) evaluated the studies according to the PICOS criteria, with any disagreements resolved through discussion with a third reviewer (F.L.).

Full texts of the preliminarily selected articles were then obtained for detailed review. Studies were included if they provided data for meta-analysis on the prevalence of targeted outcomes—periodontal diseases, dental caries, tooth wear, and orofacial pain—in overweight and/or obese individuals aged ≥ 55 years, compared to their non-overweight/obese counterparts of the same age. Only studies reporting odds ratios (OR), risk ratios (RR), rate ratios, 95% confidence intervals (CIs), or data permitting the calculation of these measures were included.

Authors of articles with incomplete data were contacted via email to request additional details, such as odds ratios, risk ratios, or two-by-two tables. A second email was sent if no response was received after two weeks, allowing another two weeks for a reply.

### Data extraction and quality assessment

Using a predetermined form, the following data were extracted: first author, year of publication, paper title, country, study design, population type, groups and sample sizes, age and sex distribution, overweight and obesity indices, measures of association, and adjustment or matching factors.

Quality assessment was performed using adapted versions of the Newcastle–Ottawa scale (NOS) for cohort and cross-sectional studies. Studies were classified as “very good” (9-10 points), “good” (7–8 points), “satisfactory” (5–6 points), or “unsatisfactory” (0–4 points). The evaluation criteria included sample representativeness, sample size, response rate, reliability of the screening tool, adjustment for potential confounders via subgroup or multivariable analysis, outcome assessment, and appropriate statistical test usage.

### Statistical methods

Meta-analysis was conducted using Stata v. 18 (StataCorp, College Station, Texas USA) (StataCorp L, 2024). Effect measures were expressed as odds ratios (ORs) and relative risks (RRs).

Heterogeneity across studies was evaluated using the *I*^2^ statistic, which quantifies the proportion of total variation attributable to between-study variance, and the DerSimonian and Laird Q test, with a *p*-value of < 0.1 indicating statistical significance. To pool effect size estimates for the association between overweight/obesity and oral health problems, each study was weighted by the inverse of its variance. A fixed-effects model was applied when heterogeneity was negligible; otherwise, a random-effects model was used.

Publication bias was visually assessed using a funnel plot and further examined with the Trim-and-Fill method to estimate the number of potentially missing or unpublished studies and their impact on the pooled effect size. Additionally, Begg’s rank correlation test and Egger’s regression test were used to statistically detect the presence of small-study effects.

## Results

### Study selection

**Fig. 1:**
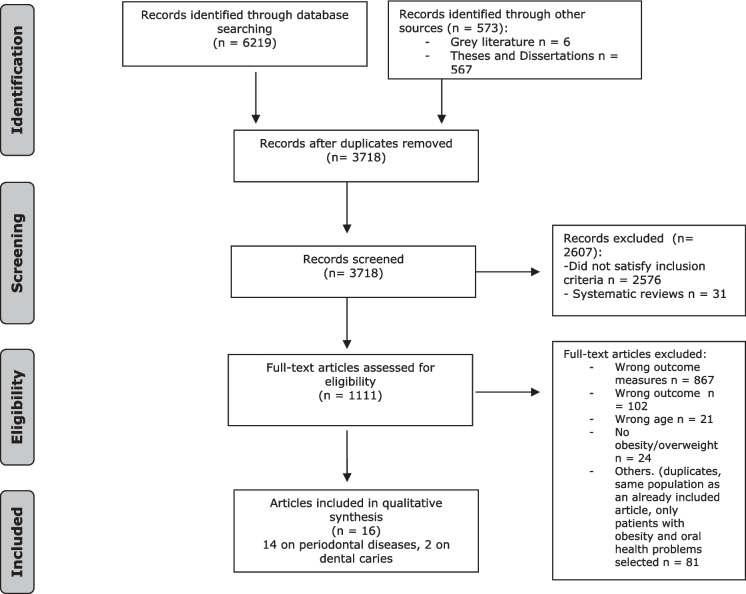
PRISMA flow diagram. Abbreviations: n, numbers

The literature search identified 6219 references across multiple databases: 1723 from PubMed, 2943 from Embase.com, 917 from CINAHL, and 636 from Web of Science. Additionally, 573 records were retrieved from grey literature (6 records) and theses/dissertations (567 records). After removing duplicates, 3718 unique references remained. Following the initial screening, 2607 studies were excluded, leaving 1111 articles for full-text review. Following a detailed review, 1095 articles were further excluded, resulting in 16 studies eligible for data extraction. The search and selection process are summarized in Fig. 1.

### Study characteristics

Among the 16 included studies, 14 examined periodontal diseases and 2 investigated dental caries. No eligible studies were identified for tooth wear or orofacial pain. Table 1 summarizes the characteristics of the periodontal disease studies, most of which were cross-sectional (*n* = 11), with three cohort studies.

**Table 1:** Characteristics of included studies evaluating the association between overweight/obesity and periodontal diseases in adults aged 55 and older

Author (year)	Country	Source of data	Total* N*	*N* of cases	Age range	Age mean (SD)	OR (95%CI)	Adjusting or matching variables	Men/women	Outcome
Al Zahrani et al. 2003 [2]	USA	The third National Health and Nutrition Examination Survey (NHANES III)	Overweight: 1239 Obese: 745 Total: 1984	Overweight: 284 Obesity: 162 Total: 446	60–90	-	1.20 (0.94, 1.46)	Sex, race, smoking, poverty index, education, diabetes, time since last visit to dentist	-	Periodontal diseases
Chen et al. 2021 [14]	Taiwan	Longitudinal Health Insurance Database 2010	Total subjects: 860, 270 obese, 590 non-obese	-	≥ 65	-	1.98 (1.22, 3.22)	-	-	Periodontal diseases
Han et al. 2010 [32]	South Korea	Sihwa-Banwol Environnemental Health Cohort Baseline data from the health cohort	-	-	≥ 55	-	1.38 (0.58, 3.33)	Age (continuous), sex, monthly family income, smoking, drinking, the frequency of daily teeth brushing and physical activity	-	Periodontitis
Iwasaki et al. 2019 [38]	Japan	Tosa Longitidunal Aging Study	Total subjects: 179, 56 obese, 123 non-obese	Obese subjects, EWP definition: Not severe: 23 Severe: 33 CDC/AAP definition: Not severe: 36 Severe: 20 Non obese subjects, EWP definition: Not severe: 55 Severe: 68 CDC/AAP definition: Not severe: 94 Severe: 29	≥ 75	80.1 (not noted)	1.80 (0.91, 3.58)	-	62/117	Periodontitis
Jimenez et al. 2012 [39]	USA	Health Professionals Follow-Up Study (HPFS)	-	BMI 25–29.9: 578 BMI > 30: 135 BMI < 24.9: 532	≥ 65	-	1.17 (1.05–1.28)	Smoking, race (White/Black/Asian/other), dental profession (yes/no), physical activity (quintiles), fruit and vegetable intake (quintiles), alcohol consumption (g/day: 0, 0.1–4.9, 5–14.9, 15–29, 30 +), diabetes status at baseline (yes/no)	All men	Self-reported periodontitis
Kitagawa et al. (2017) [45]	Japan	Public health centers	Total subjects: 13,639 obese, 236	Obese with CPI 3–4: 194 Non obese with CPI 3–4: 8500	60	60	5.35 (3.83–7.48)	-	115/121	CPI 3–4
Lee et al. 2014[50]	South Korea	Seongju-gun Public Health Center	78	Obese for ≥ 6yrs: CPI 3 = 20, CPI 4 = 9 Obese for < 6yrs: CPI 3 = 12, CPI 4 = 3 Total with CPI 3–4 = 44	≥ 60	-	3.09 (1.49–4.69)	-	-	Periodontal disease
Linden et al. 2007 [51]	UK	Participants of the Prospective Epidemiological Study of Myocardial Infarction (PRIME)	Overweight: 501 Obese: 171 Total: 672	Overweight: Low threshold = 180, high threshold = 47, obese: low threshold = 97, high threshold = 30	60–70	Overweight: 64.4 (2.9) Obese: 64.0 (2.9)	1.37 (1.01, 1.73)	Age, smoking, diabetes, years in education, socio-economic status, dental attendance, and toothbrushing frequency	All men	Periodontitis (low and high threshold)
Minagawa et al. 2015 [54]	Japan	Participants from the Niigata study	Total = 234 *N* abdominal obesity = 134	Moderate periodontitis: 116 Severe periodontitis: 2	80	80 (0)	1.12 (0.62, 2.06)	Gender, income, education, smoking status, pattern of visits to a dentist, brushing frequency, exercise habits, and dietary energy and food intake	-	Moderate and severe periodontitis
Munoz torres et al. 2014 [57]	Puerto Rico	Participants who enrolled in the Puerto Rican Elderly Dental Health Study (PREDHS)	High WC: 96 High WHR: 92	High WC, Severe periodontitis: 21 Moderate periodontitis: 39 High WHR, Severe periodontitis: 17 Moderate periodontitis: 43	≥ 70	High WC: 78.3 (6.0) High WHR: 78.7 (6.0)	1.66 (0.27, 3.04)	Model 1: age, gender Model 2: Model 1 + additionally adjusted for smoking, education, diabetes status, physical activity, total fruit, and vegetable intake	High WC: 25/71 High WHR: 28/64	Periodontitis (moderate and severe)
Oikarinen et al. 2014 [59]	Finland	Sample taken from population belonging to the Geriatric Multidisciplinary Strategy for the Good Care of Older People (GeMS) study	BMI 25 to < 30: *N* = 64 ≥ 30: *N* = 32	25 to < 30, *N* = 34 ≥ 30, *N* = 19	≥ 75	25 to 30: 81.1 (4.2) ≥ 30: 80.1 (3.3)	0.70 (0.40, 1.20)	-	25 to < 30: 24/40 ≥ 30: 17/15	Presence of PD of ≥ 4 mm
Rivas-Tumanyan et al. [69]	Puerto Rico	Participants who enrolled in the Puerto Rican Elderly Dental Health Study (PREDHS)	182 BMI ≥ 25, *N* = 118 BMI < 25 = 64	BMI ≥ 25, *N* = 90 BMI < 25, *N* = 49	-	-	1.02 (0.50, 2.08)	-	-	Severe periodontitis
Teixeira et al. 2020 [88]	Brazil	Participants from the major “Analysis of Diet and Lifestyle for Cardiovascular Prevention in Seventh-Day Adventists” study	Total = 191 *N* overweight = 65 *N* obese = 37	Mild/moderate periodontitis, Not overweight/obese: 19 Overweight: 16 Obese: 10 Severe periodontitis, not overweight/obese: 7 Overweight: 6 Obese: 8	≥ 55	-	1.20 (0.76, 1.90)	-	-	Moderate and severe periodontitis
Yang et al. 2023 [100]	China	Data from the 2009–2014 cycle of the National Health and Nutrition Examination survey	4481 subjects were analyzed	-	-	-	0.76 (0.19, 1.33)	-	-	Periodontitis

Abbreviations: *N*, numbers; *SD*, standard deviation; *OR*, odds ratio; *CI*, confidence interval; *EWP*, European Workshop in Periodontology group C; *CDC/AAP*, Centers for Disease Control/American Academy of Periodontology; *BMI*, body mass index; *CPI*, Community Periodontal Index; *WC*, waist circumference; *WHR*, waist-to-hip ratio

The periodontal disease studies were conducted in Asia (*n* = 6), North America (*n* = 5), Europe (*n* = 2), and South America (*n* = 1). The reported prevalence of periodontal diseases ranged from 6.5% [51] to 59.4% [59].

For dental caries (Table 2), Rodrigues et al. [70] reported a prevalence of 15.8%, whereas Sheng et al. [77] did not specify a prevalence rate. Both studies were cross-sectional in design.

**Table 2:** Characteristics of included studies evaluating the association between overweight/obesity and caries in adults aged 55 and older

Author (year)	Country	Source of data	Total *N*	*N* of cases	Age range	Age mean (SD)	OR (95%CI)	Adjusting or matching variables	Men/women	Outcome
**Rodrigues et al. 2012** [70]	Brazil	Group for the Elderly Interdisciplinary Geriatrics and Gerontology Program of the Fluminense Federal University, RJ, Brazil	Normal BMI = 5 Altered BMI = 20	Normal BMI, *N* = 2 Altered BMI, *N* = 3	≥ 60	-	0.19 (0.02–1.90)	-	-	Unsatisfactory number of decayed teeth
**Sheng et al. 2018** [77]	China	Population data were collected from 13 districts in Southwest China from September 2013 to January 2015	Obese: 24, Overweight: 103	-	≥ 60	-	1.05 (0.56, 1.54)	-	-	Dental caries

Abbreviations: *N*, numbers; *SD*, standard deviation; *OR*, odds ratio; *CI*, confidence interval; *BMI*, body mass index

### Quality assessment

The quality assessment of the included studies is summarized in Table 3. Of the 16 studies, most were rated as “good” or “very good,” while Rodrigues et al. [70] received a “satisfactory” rating.

**Table 3:** Quality assessment of included studies (Newcastle–Ottawa scale)

	Selection (Max. 5)				Comparability (Max. 2)	Outcome (Max. 2)	Statistical test (Max.2)		
Study	**Q1**	**Q2**	**Q3**	**Q4**	**Q5**	**Q6**	**Q7**	**Subtotal**	**Quality category**
Al Zahrani et al. 2003 [2]	+	+	+	+ +	+ +	+ +	+	10	Very good
Chen et al. 2021 [14]	+	+	+	+ +	+ +	+	-	8	Good
Han et al. 2010 [32]	-	-	+	+ +	+ +	+ +	+	8	Good
Iwasaki et al. 2019 [38]	+	+	+	+ +	–	+ +	-	7	Good
Jimenez et al. 2012 [39]	+	+	+	+ +	+ +	+	+	9	Very good
Kitagawa et al. 2017 [45]	+	+	-	+ +	+ +	+ +	+	9	Very good
Lee et al. 2014 [50]	+	+	+	+ +	–	+ +	+	8	Good
Linden et al. 2007 [51]	-	+	+	+ +	+ +	+ +	+	9	Very good
Minagawa et al. [54]	+	+	+	+ +	+ +	+ +	+	10	Very good
Muñoz Torres et al. 2014 [57]	+	+	+	+ +	+ +	+ +	+	10	Very good
Oikarinen et al. 2014 [59]	+	+	+	+ +	–	+ +	+	8	Good
Rivas-Tumanyan et al. [69]	+	+	+	+ +	+ +	+ +	+	10	Very good
Rodrigues et al. 2010 [70]	+	-	-	+ +	–	+ +	+	6	Satisfactory
Sheng et al. 2018 [77]	+	-	+	+ +	–	+ +	+	7	Good
Teixeira et al. 2020 [88]	+	+	+	+ +	–	+ +	-	7	Good
Yang et al. 2023 [100]	+	+	-	+ +	+ +	+ +	+	9	Very good

Max: maximum score

Q1, representativeness of the sample; Q2, sample size; Q3, non-respondents; Q4, ascertainment of the screening tool; Q5, potential confounders investigated by subgroup/multivariable analysis; Q6, assessment of outcome; Q7, statistical test

Scoring criteria: + , criterion met (1 point); +  + , criterion strongly met (2 points); -, criterion not met (0 points); –, not addressed or failed entirely (0 points)

Quality categories: Very good, 9–10 points; Good, 7–8 points; Satisfactory, 5–6 points

In the “selection” domain (Q1–Q4), the majority of studies scored 4 or 5 out of 5 points. However, two studies [32, 70] scored three points due to limitations in sample representativeness (Q1) and sample size justification (Q2). Kitagawa et al. [45], Rodrigues et al. (Rodrigues Junior et al., 2012) and Yang et al. (Yang et al., 2023) also lacked data on non-respondents (Q3). All studies scored full points for using validated screening tools (Q4).

In the “comparability” domain (Q5), six studies [38, 50, 59, 70, 77, 88] did not adequately investigate potential confounders through subgroup or multivariable analysis, making it the least favorably scored domain.

In the “outcome” domain (Q6), most studies achieved the highest possible scores, except Jimenez et al. [39] which relied on self-reported outcomes.

In the “statistical test” domain (Q7), two studies [38, 88] scored poorly due to unclear or inappropriate statistical methods.

Using the Newcastle–Ottawa scale, studies scoring 9–10 points were categorized as “very good,” 7–8 as “good,” and 5–6 as “satisfactory.” None fell into the “unsatisfactory” category (0–4 points).

### Synthesis of results

**Fig. 2:**
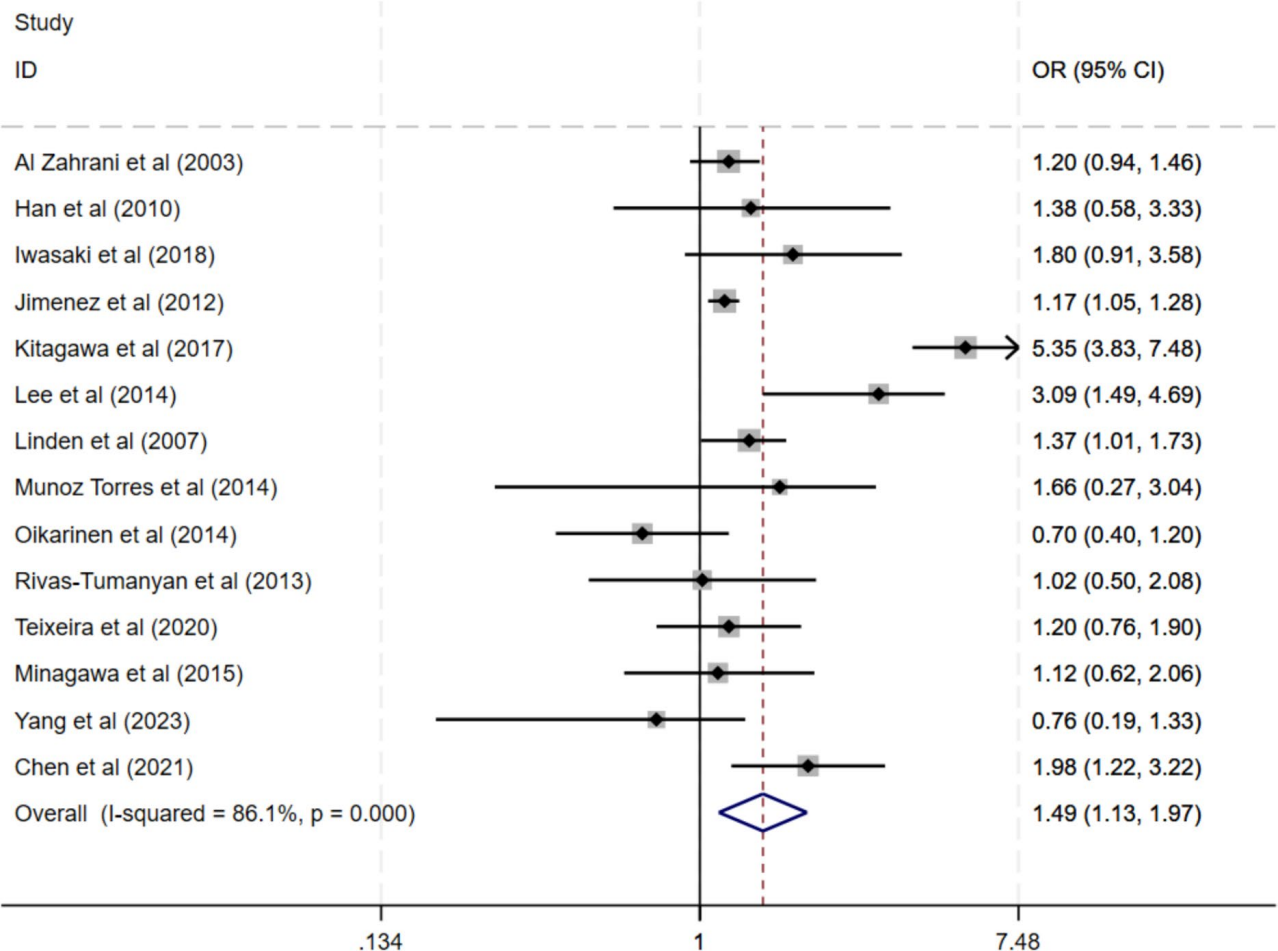
Forest plot illustrating the association between overweight/obesity and periodontal diseases in adults aged 55 and older. Abbreviations: OR, odds ratio; CI, confidence interval

A random-effects meta-analysis revealed a significantly higher likelihood of periodontal diseases in elderly individuals with overweight/obesity, with a pooled OR of 1.49 (95% CI 1.13–1.97) (Table 4, Fig. 2).

**Table 4:** Random-effects pooled odds ratios (ORs) and 95% confidence intervals (CIs) for the association between overweight/obesity and periodontal diseases across subgroups

Groups	Number of studies	OR (95% CI)	***I***^2^ (%)	***P***-value for heterogeneity
All studies	14	1.49 (1.13–1.97)	86.1	< 0.001
**Design**				
Cross-sectional	11	1.45 (0.97–2.16)	87.2	< 0.001
Cohort	3	1.49 (1.001–2.22)	65.0	0.06
**Continent**				
Asia	6	2.21 (1.28–3.81)	83.0	< 0.001
Europe	2	1.02 (0.53–1.96)	78.8	0.03
S.A	1	1.20 (0.63–1.77)	-	-
N.A	5	1.17 (1.07–1.28)	0.0	0.87
N.A and S.A as one subgroup	6	1.17 (1.07, 1.28)	0.0	0.94
**Sex**				
Male	2	1.20 (1.07–1.34)	11.9	0.29
Mixed	12	1.52 (1.02–2.28)	85.9	< 0.001

Abbreviations: *OR* odds ratio, *CI* confidence intervals, *N.A* North America, *S.A* South America

Subgroup analysis showed a stronger association in cross-sectional studies (OR = 1.45, 95% CI 0.97–2.16, *I*^2^ = 82.2%, *P* < 0.001) than in cohort studies (OR = 1.49, 95% CI 1.001–2.22, *I*^2^ = 65.0%, *P* = 0.06).

Geographically, Asian studies showed the strongest association (OR = 2.21, 95% CI 1.28–3.81, *I*^2^ = 83.0%, *P* < 0.001). North American (N.A.) studies showed a weaker but significant association (OR = 1.17, 95% CI 1.07–1.28, *I*^2^ = 0.0%, *P* = 0.87). South American (S.A.) studies had an OR of 1.20 (95% CI 0.63–1.77) but were limited by a single study. European studies showed no significant association (OR = 1.02, 95% CI 0.53–1.96, *I*^2^ = 78.8%, *P* = 0.03).

Sex-based analysis showed a stronger association in mixed-sex studies (OR = 1.52, 95% CI 1.02–2.28, *I*^2^ = 85.9%, *P* < 0.001) compared to male-only studies (OR = 1.20, 95% CI 1.07–1.34, *I*^2^ = 11.9%, *P* = 0.29).

Due to the limited number and inconsistent results of the two small cross-sectional studies on dental caries, a meta-analysis for this outcome was not feasible.

### Publication bias

**Fig. 3:**
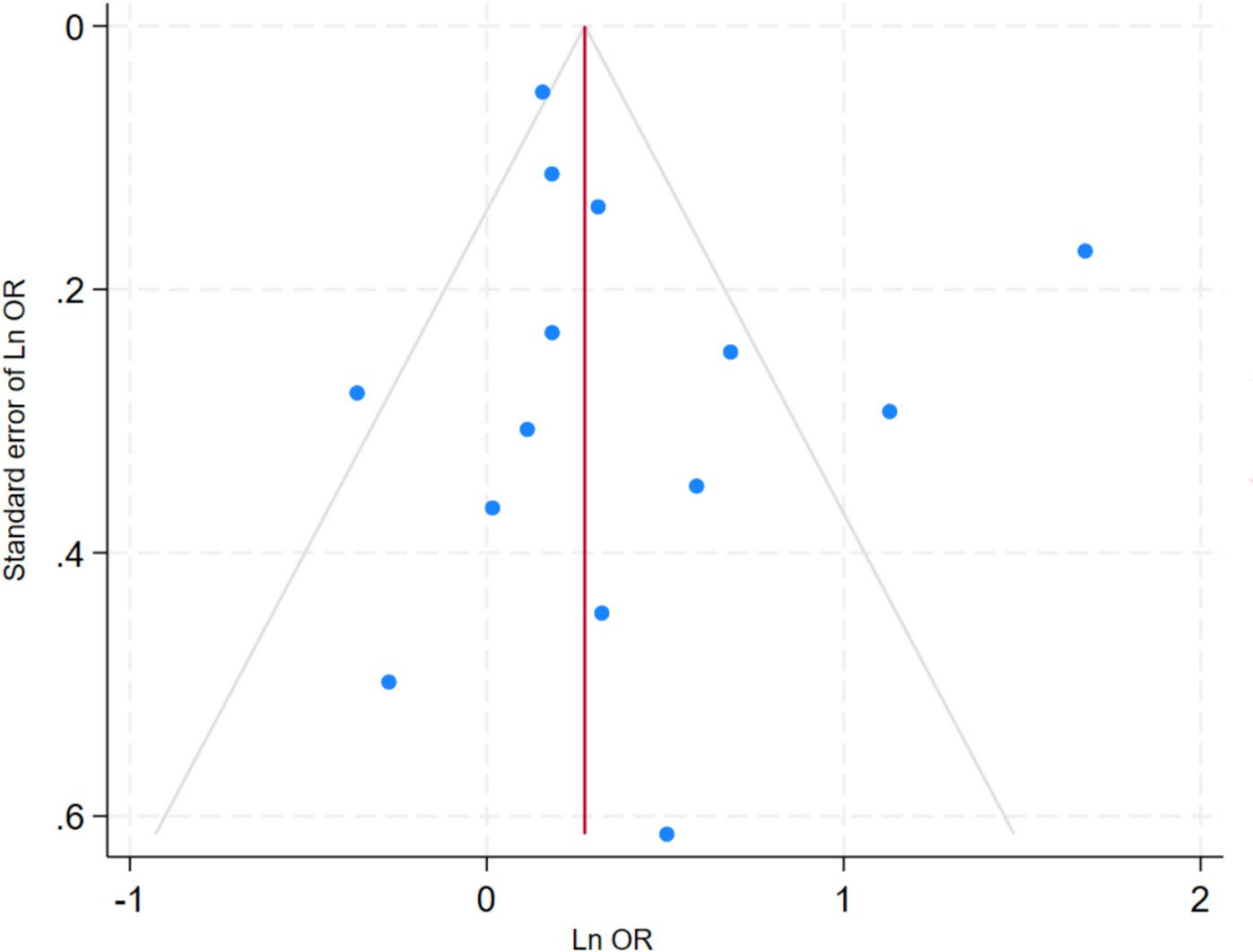
Funnel plot illustrating the association between overweight/obesity and periodontal diseases

Visual inspection of the funnel plot revealed no evidence of publication bias among the 14 studies evaluating the association between overweight/obesity and periodontal diseases. The plot appeared symmetrical, and the Trim-and-Fill procedure did not impute any additional studies, suggesting no missing data due to publication bias. These findings were further supported by Egger’s regression test (*β*₁ = –0.50, SE = 1.11, *P* = 0.65) and Begg’s rank correlation test (Kendall’s score = 5.0, SE = 18.26, *P* = 0.82), both indicating a low likelihood of small-study effects (Fig. 3).

## Discussion

The high prevalence of overweight and obesity among the elderly population underscores the importance of understanding its implications for oral health. This comprehensive review and meta-analysis address a critical gap in existing research by systematically analyzing global data on oral health issues—including periodontal diseases, dental caries, tooth wear, and orofacial pain—among overweight and obese older adults. To our knowledge, this is the first study to comprehensively evaluate these associations in this population.

Defining what constitutes “elderly” remains a challenge, as criteria vary widely across studies and sources. Some define the elderly as individuals aged 50 and older, while others extend this threshold to 70 years [11, 28]. Numerous studies have highlighted a rising incidence of multimorbidity among middle-aged and older adults, even starting in working-age populations [35, 72]. Multimorbidity, in turn, has been shown to negatively impact oral health outcomes [27]. Considering these factors, we chose to include participants aged 55 and above in our review. This cutoff provides a balanced approach by capturing individuals at increased risk for obesity-related complications while maintaining consistency with the age criteria used in comparable studies [17, 88].

We undertook an extensive search of multiple databases, supplemented by grey literature, and included cross-sectional, case–control, cohort studies, and clinical trials to maximize the number of eligible studies. However, we restricted our review to English-language publications to reduce potential translation errors. The overall methodological quality of included studies was relatively high. Common limitations included insufficient adjustment for potential confounders in six studies (Q5) [38, 50, 59, 70, 77, 88], making the “comparability” the weakest domain in the quality assessment. Additional deficiencies were noted in study selection procedures (Han et al., 2010; Kitagawa et al., 2017; Linden et al., 2007; Rodrigues Junior et al., 2012; Sheng et al., 2018; Yang et al., 2023), unclear statistical testing in two studies [38, 88], and limited outcome assessment in Jimenez et al. [39].

Our random-effects meta-analysis demonstrated that elderly individuals with overweight or obesity have a significantly higher likelihood of developing periodontal diseases (pooled OR = 1.49, 95% CI 1.13–1.97), despite high heterogeneity across studies (*I*^2^ = 86.1%, *P* < 0.001). This heterogeneity likely reflects differences in study populations, diagnostic criteria, and methodologies. Nevertheless, the consistency of positive associations across diverse settings strengthens the evidence for a genuine relationship between overweight/obesity and periodontal diseases in this age group. Notably, the results of two cohort studies [14, 39] and three cross-sectional studies [45, 50, 51] aligned closely with the pooled estimate, despite differences in geographic location and disease definitions. These findings emphasize the need for standardized diagnostic criteria and study methodologies in future research to improve comparability and validate the observed association.

The association between obesity, aging, and periodontal diseases can be attributed to several interconnected biological and pathological mechanisms. The synergistic effects of obesity and aging amplify endothelial oxidative stress and increase the expression of nicotinamide adenine dinucleotide phosphate (NADPH) oxidase, leading to elevated superoxide levels from both NADPH oxidases and mitochondrial sources. These superoxide radicals combine with endothelium-derived nitric oxide to form peroxynitrite, reducing nitric oxide bioavailability and contributing to endothelial dysfunction during aging and obesity [8, 26, 91]. Interestingly, nitrate has been shown to protect against—and potentially reverse—endothelial dysfunction induced by periodontitis, by restoring nitrite and, consequently, nitric oxide levels [23].

Obesity-related comorbidities, such as hypertension, may also contribute to capillary rarefaction observed in aging [86]. Additionally, the interaction between aging and obesity may impair the synthesis of vasoactive arachidonic acid metabolites [93]. Among these, prostaglandin E2, a potent stimulator of alveolar bone resorption, plays a significant pathogenic role in periodontal breakdown [12].

Obesity is characterized by chronic low-grade inflammation in adipose tissue, marked by increased macrophage infiltration, altered immune cell phenotypes, and a proinflammatory secretome. This results in elevated levels of circulating inflammatory mediators [21, 85]. Growing evidence suggests that aging intensifies this adipose tissue inflammation [80, 93], further contributing to secondary conditions such as type 2 diabetes mellitus [41], which are also associated with increased periodontal risk.

Moreover, both aging and obesity are linked to dysbiosis of the oral and gut microbiome [10, 78]. A common microbial pattern includes increased abundance of Firmicutes and a reduction in Bacteroides [53, 71, 89], which has been implicated in systemic and periodontal inflammation. In addition, cellular senescence in vascular tissues may contribute to the vascular pathologies associated with both aging and obesity [25, 87, 99]. Emerging evidence also suggests that cellular senescence may play a key role in the pathophysiology of periodontal tissue degradation [3].

Sex-specific hormonal changes, particularly the decline in estrogen during menopause, may further influence the relationship between obesity and periodontal disease in older women. Estrogen plays a protective role by maintaining alveolar bone density, modulating immune responses, and supporting periodontal tissue integrity. Its decline is associated with elevated systemic proinflammatory cytokine levels and increased susceptibility to periodontitis [64]. When combined with obesity, a condition already marked by metabolic dysregulation and inflammation, this hormonal shift may exacerbate periodontal tissue destruction. Additionally, estrogen deficiency has been associated with reduced microbial diversity and increased pathogenic load in both the oral and gut microbiome [6, 94], potentially amplifying obesity-related dysbiosis. These findings suggest that postmenopausal women with obesity may represent a particularly high-risk subgroup for periodontal disease progression, highlighting the need for targeted monitoring and personalized prevention strategies.

Importantly, lifestyle interventions may help interrupt the inflammatory cascade linking obesity with periodontal deterioration. In older adults with obesity, moderate caloric restriction combined with regular aerobic and resistance exercise improves metabolic function and reduces systemic inflammation, specifically lowering levels of C-reactive protein (CRP) and interleukin-6 (IL-6), both of which are implicated in periodontal tissue destruction [19].

Simultaneously, adopting an anti-inflammatory dietary pattern, such as a Mediterranean-style or predominantly plant-based diet rich in fruits, vegetables, whole grains, legumes, nuts, olive oil, and fatty fish, has been shown to inhibit NF-κB signaling, promote the anti-inflammatory M2 macrophage phenotype, and enhance endothelial function [30]. On a molecular level, key bioactive nutrients such as polyphenols, omega-3 fatty acids, and dietary fiber can further suppress obesity-related inflammation by downregulating MAP-kinase pathways, reducing endoplasmic reticulum stress, and stimulating the secretion of anti-inflammatory adipokines [84]. Incorporating these lifestyle modifications into the dental care and counseling of adults aged 55 and older may offer a practical strategy to slow or prevent periodontal disease progression in overweight and obese individuals.

Our subgroup analyses revealed notable regional differences in the association between overweight/obesity and periodontal disease. The strongest association was observed in studies from Asia (OR = 2.21, 95% CI 1.28–3.81), whereas European studies showed a weaker and non-significant association (OR = 1.02, 95% CI 0.53–1.96). One potential explanation for this discrepancy lies in the higher prevalence of metabolic disorders and systemic inflammation in Asian populations, which may be linked to a greater propensity for visceral fat accumulation compared to European populations [55, 83]. Visceral fat is more metabolically active and pro-inflammatory, potentially exacerbating periodontal disease.

Genome-wide association studies have also identified ethnicity-specific genetic variants affecting fat distribution. In particular, genes associated with embryonic development and metabolic regulation are more frequently observed in Asians, possibly predisposing them to long-term metabolic vulnerabilities [83]. In contrast, Europeans tend to accumulate more subcutaneous fat, which is less metabolically active and may pose a lower risk for systemic inflammation and its oral health consequences [83]. These findings point to the need for further investigation into ethnicity-specific genetic and metabolic factors to better explain regional disparities and support the development of population-tailored prevention strategies. Cultural differences in dietary habits and oral hygiene practices may also contribute to regional variation. In Europe, substantial heterogeneity among studies may reflect variation in healthcare systems, public health initiatives, and levels of oral health awareness across different countries. Additional factors such as socioeconomic disparities, differences in adherence to oral hygiene recommendations, and unequal access to specialized dental care may further explain this heterogeneity.

Regarding sex differences, studies including both men and women reported a stronger association (OR = 1.52, 95% CI 1.02–2.28) than male-only studies (OR = 1.20, 95% CI 1.07–1.34). However, heterogeneity was substantially higher in the mixed-sex group (*I*^2^ = 85.9%) compared to male-only studies (*I*^2^ = 11.9%). This greater variability may reflect sex-specific differences in hormonal regulation, body composition, lifestyle behaviors, and the metabolic consequences of obesity. The decline in estrogen levels during menopause, as discussed earlier, may intensify periodontal risk in women through both systemic and local mechanisms. In contrast, men and women differ in fat distribution, inflammation profiles, and susceptibility to metabolic disorders, all of which may modulate oral disease outcomes differently. These findings underscore the importance of incorporating sex-stratified analyses in future research to better understand and address these disparities. Developing tailored, sex-specific preventive and therapeutic approaches will be crucial for effective periodontal disease management in aging populations.

Two systematic reviews have previously noted a secondary peak in dental caries prevalence around the age of 70 [7, 42]. In contrast, our analysis identified only two studies on caries among overweight or obese older adults, both yielding inconsistent findings. These cross-sectional studies lacked the ability to assess incidence or determine temporal relationships between obesity and caries [96]. Moreover, longitudinal studies involving older adults with obesity often face elevated dropout rates due to cognitive decline, functional limitations, and increased mortality risk.

A 10-year follow-up study by Edman et al. found that caries occurrence in older adults is not directly attributable to aging per se, but rather to increasing dependency on care, a factor that typically escalates with age [18]. Interestingly, Rodrigues et al. reported a lower number of decayed teeth among overweight/obese individuals compared to those with normal BMI [70], but the limited sample size and cross-sectional design preclude causal inference and limit generalizability. Conversely, Chala et al. found a U-shaped association between BMI and caries, suggesting that both underweight and overweight statuses are linked to increased caries risk [49]. Given this variability, future clinical studies should move beyond global indices such as DMFT and instead evaluate lesion-specific characteristics—such as location, severity, and activity, tailored to the elderly obese population.

Obesity imposes a pro-nociceptive state in individuals, which may facilitate the pain processes, and has been shown to be associated with some types of chronic pain [60, 79]. A machine-learning study by Lee et al. identified both BMI and age as key predictors of temporomandibular disorders [49]. Despite these findings, no studies specifically addressing orofacial pain or tooth wear in elderly overweight/obese populations were identified in our review. A study by Ashour et al. found no significant differences in tooth wear prevalence between obese and non-obese individuals [4], aligning with Salas et al., who reported no correlation between obesity and dental erosion in a Brazilian cohort [73]. The lack of focused studies on this demographic highlights a substantial gap in understanding these relationships, which may reflect either insufficient research or a weak direct connection between obesity and these outcomes. Further longitudinal studies are needed to elucidate the potential risks of tooth wear and orofacial pain in elderly individuals with obesity, enabling a deeper understanding of these issues in this vulnerable population.

The strengths of this study include its strict adherence to PRISMA guidelines, a well-defined PICOS criteria, and a comprehensive systematic literature search independently conducted by two reviewers. Nonetheless, several limitations should be acknowledged. Considerable heterogeneity in study designs, methodologies, and populations limits the generalizability of the findings, particularly given the variation in definitions of overweight/obesity and periodontal diseases across studies. Small sample sizes in several included studies may have reduced statistical power and introduced bias into effect estimates. Restricting the review to English-language publications may have excluded relevant evidence from non-English sources. Furthermore, the inclusion of both studies with and without control groups, while consistent with previous research [47, 48, 75] and enriching the dataset, also increased methodological variability. These issues underscore the urgent need for future research to apply standardized diagnostic criteria for both obesity and oral health outcomes. Robust study designs, such as longitudinal cohort studies with larger and more ethnically diverse populations, are essential to enhance reliability and improve comparability of results. Additionally, critical evidence gaps persist regarding caries, tooth wear, and orofacial pain in overweight and obese older adults. Addressing these gaps would offer a more comprehensive understanding of the complex interactions between obesity, aging, and oral health, ultimately guiding the development of targeted preventive strategies and interventions.

From a clinical perspective, the findings underscore the importance of integrating weight status into routine dental assessments for adults aged 55 and older. Overweight and obese elderly patients, particularly those with additional risk factors such as diabetes, smoking, or reduced manual dexterity, may require closer monitoring for oral health problems, including periodontal disease progression. Current evidence on optimal recall intervals remains limited and heterogeneous. Observational data indicate that oral health indices such as DMFT (decayed, missing, filled teeth), DT (decayed teeth), and CPI (Community Periodontal Index) are reliable predictors of individualized recall interval needs, with poorer scores associated with shorter intervals, independent of age, sex, or comorbidities [34]. However, there is currently no high-quality research specifically addressing recall intervals tailored to overweight and obese older adults, highlighting an urgent need for trials in this subgroup. Preventive interventions could include personalized oral hygiene instructions, dietary counseling aimed at reducing systemic inflammation, and interdisciplinary collaboration with physicians for weight management. Furthermore, public health strategies should integrate oral health promotion within broader obesity prevention programs targeting this vulnerable population.

Future studies should aim to overcome current methodological limitations by employing large-scale, longitudinal cohort designs with sufficient sample sizes based on power calculations to ensure reliable effect estimates. Standardized and universally accepted diagnostic criteria for obesity and oral health problems, including periodontal diseases, are essential to improve comparability across studies. Long follow-up periods are recommended to clarify temporal relationships and causality, particularly in understanding how obesity and aging jointly contribute to periodontal disease progression. Future research should also incorporate sex-stratified analyses, as well as microbiome and inflammatory biomarker profiling, to better elucidate underlying mechanisms. Randomized controlled trials evaluating the effect of weight loss and anti-inflammatory dietary interventions on oral health outcomes in older adults would provide valuable evidence to guide preventive and therapeutic strategies.

## Conclusions

This systematic review examined the relationship between overweight/obesity and various oral health outcomes, including periodontal diseases, dental caries, tooth wear, and orofacial pain, in adults aged 55 and older. Our findings demonstrate a significant association between overweight/obesity and increased prevalence of periodontal disease in this population. Evidence regarding dental caries is limited and inconsistent, and data on tooth wear and orofacial pain are virtually absent. Future research should prioritize well-designed, longitudinal studies to clarify biological and behavioral mechanisms underlying obesity-related periodontal breakdown and to explore understudied outcomes. Given the substantial social, economic, and psychological burden of oral diseases in older adults, developing targeted preventive strategies and integrating oral health into obesity management programs is imperative. Addressing these evidence gaps will enhance clinical decision-making, guide public health policies, and ultimately improve the quality of life for this growing, high-risk demographic.

## Data Availability

The datasets analyzed during this study are available from the corresponding authors upon reasonable request.

## References

[1] Alshakhs M, Jackson B, Ikponmwosa D, Reynolds R, Madlock-Brown C. Multimorbidity patterns across race/ethnicity as stratified by age and obesity. Sci Rep. 2022;12:9716. 10.1038/s41598-022-13733-w. 35690677 10.1038/s41598-022-13733-w PMC9188579

[2] Al-Zahrani MS, Bissada NF, Borawski EA. Obesity and periodontal disease in young, middle-aged, and older adults. J Periodontol. 2003;74:610. 10.1902/jop.2003.74.5.610. 12816292 10.1902/jop.2003.74.5.610

[3] Aquino-Martinez R. The emerging role of accelerated cellular senescence in periodontitis. J Dent Res. 2023;102:854. 10.1177/00220345231154567. 36908187 10.1177/00220345231154567

[4] Ashour AA, Fahmi MK, Mohamed RN, Basha S, Binmadi N, Enan ET, Basalim A, Al Qahatani A. Association between gastric reflux, obesity and erosive tooth wear among psychiatric patients. Medicine (United States). 2022;101:28923. 10.1097/MD.0000000000028923. 10.1097/MD.0000000000028923 PMC9281915 35363217

[5] Baniasadi K, Armoon B, Higgs P, Bayat AH, MohammadiGharehghani MA, Hemmat M, Fakhri Y, Mohammadi R, Fattah Moghaddam L, Schroth RJ. The association of oral health status and socio-economic determinants with oral health-related quality of life among the elderly: a systematic review and meta-analysis. Int J Dent Hyg. 2021;19:12489. 10.1111/idh.12489. 10.1111/idh.12489 33523593

[6] Bayardo-González RA, Peña-Rodríguez M, Pereira-Suárez AL, Rubio-Sánchez AX, García-Chagollán M, Valenzuela-Orozco DN, del Lizarazo-Taborda MR, Mora-Mora J, Vega-Magana N. Insights into estrogen impact in oral health & microbiome in COVID-19. BMC Microbiol. 2024;24:32. 10.1186/s12866-023-03149-5. 38245675 10.1186/s12866-023-03149-5 PMC10799413

[7] Borg-Bartolo R, Roccuzzo A, Molinero-Mourelle P, Schimmel M, Gambetta-Tessini K, Chaurasia A, Koca-Ünsal RB, Tennert C, Giacaman R, Campus G. Global prevalence of edentulism and dental caries in middle-aged and elderly persons: a systematic review and meta-analysis. J Dent. 2022;127:104335. 10.1016/j.jdent.2022.104335. 36265526 10.1016/j.jdent.2022.104335

[8] Bourgoin F, Bachelard H, Badeau M, Mélançon S, Pitre M, Larivière R, Nadeau A. Endothelial and vascular dysfunctions and insulin resistance in rats fed a high-fat, high-sucrose diet. Am J Physiol Heart Circ Physiol. 2008;2008:295. 10.1152/ajpheart.00516.2008. 10.1152/ajpheart.00516.2008 18599593

[9] Bramer WM, Giustini D, De Jong GB, Holland L, Bekhuis T. De-duplication of database search results for systematic reviews in endnote. Journal of the Medical Library Association. 2016;104:014. 10.3163/1536-5050.104.3.014. 10.3163/1536-5050.104.3.014 PMC4915647 27366130

[10] Buford TW, Carter CS, VanDerPol WJ, Chen D, Lefkowitz EJ, Eipers P, Morrow CD, Bamman MM. Composition and richness of the serum microbiome differ by age and link to systemic inflammation. Geroscience. 2018;40:257. 10.1007/s11357-018-0026-y. 29869736 10.1007/s11357-018-0026-y PMC6060185

[11] Cardoso L, Rodrigues D, Gomes L, Carrilho F. Short- and long-term mortality after bariatric surgery: a systematic review and meta-analysis. Diabetes Obes Metab. 2017;19:12922. 10.1111/dom.12922. 10.1111/dom.12922 28244626

[12] Cekici A, Kantarci A, Hasturk H, Van Dyke TE. Inflammatory and immune pathways in the pathogenesis of periodontal disease. Periodontol. 2000;2014:64. 10.1111/prd.12002. 10.1111/prd.12002 PMC4500791 24320956

[13] Chapple ILC, Milward MR, Dietrich T. The prevalence of inflammatory periodontitis is negatively associated with serum antioxidant concentrations. Journal of Nutrition. 2007;137:657. 10.1093/jn/137.3.657. 17311956 10.1093/jn/137.3.657

[14] Chen TP, Yu HC, Lin TH, Wang YH, Chang YC, Li S. Association between obesity and chronic periodontitis: a nationwide population-based cohort study in Taiwan. Medicine (United States). 2021;100:e27506. 10.1097/MD.0000000000027506. 10.1097/MD.0000000000027506 PMC8519219 34731134

[15] Dibello V, Lobbezoo F, Lozupone M, Sardone R, Ballini A, Berardino G, Mollica A, Coelho-Júnior HJ, De Pergola G, Stallone R, Dibello A, Daniele A, Petruzzi M, Santarcangelo F, Solfrizzi V, Manfredini D, Panza F. Oral frailty indicators to target major adverse health-related outcomes in older age: a systematic review. Geroscience. 2023;45:663. 10.1007/s11357-022-00663-8. 36242694 10.1007/s11357-022-00663-8 PMC9886742

[16] Dibello V, Lobbezoo F, Sardone R, Lozupone M, Castellana F, Zupo R, Pilotto A, Daniele A, Solfrizzi V, Manfredini D, Panza F. The relationship between oral health-related quality of life and body mass index in an older population from Southern Italy: the Salus in Apulia study. J Pers Med. 2023;13:1300. 10.3390/jpm13091300. 37763068 10.3390/jpm13091300 PMC10533155

[17] Dros C, Sealy MJ, Krijnen WP, Weening-Verbree LF, Hobbelen H, Jager-Wittenaar H. Oral Health and frailty in community-dwelling older adults in the northern Netherlands: a cross-sectional study. Int J Environ Res Public Health. 2022;19:7654. 10.3390/ijerph19137654. 35805314 10.3390/ijerph19137654 PMC9265776

[18] Edman K, Holmlund A, Norderyd O. Caries disease among an elderly population—a 10-year longitudinal study. Int J Dent Hyg. 2021;19:12490. 10.1111/idh.12490. 10.1111/idh.12490 33523601

[19] Ellis A, Crowe K, Lawrence J. Obesity-related inflammation: implications for older adults. J Nutr Gerontol Geriatr. 2013;32:842199. 10.1080/21551197.2013.842199. 10.1080/21551197.2013.842199 24224937

[20] Elmaleh-Sachs A, Schwartz JL, Bramante CT, Nicklas JM, Gudzune KA, Jay M. Obesity management in adults. JAMA. 2023;330:19897. 10.1001/jama.2023.19897. 10.1001/jama.2023.19897 PMC11325826 38015216

[21] Fain JN. Release of interleukins and other inflammatory cytokines by human adipose tissue is enhanced in obesity and primarily due to the nonfat cells. Vitam Horm. 2006;74:443. 10.1016/S0083-6729(06)74018-3. 17027526 10.1016/S0083-6729(06)74018-3

[22] Falter T, Hennige AM, Schulz A, Gieswinkel A, Lotz J, Rossmann H, Beutel M, Michal M, Pfeiffer N, Schmidtmann I, Münzel T, Wild PS, Lackner KJ. Prevalence of overweight and obesity, its complications, and progression in a 10-year follow-up in the Gutenberg health study (GHS). Obes Facts. 2024;17:533671. 10.1159/000533671. 10.1159/000533671 PMC10836863 37839401

[23] Fernandes D, Khambata RS, Massimo G, Ruivo E, Gee LC, Foster J, Goddard A, Curtis M, Barnes MR, Wade WG, Godec T, Orlandi M, D’Aiuto F, Ahluwalia A. Local delivery of nitric oxide prevents endothelial dysfunction in periodontitis. Pharmacol Res. 2023;188:106616. 10.1016/j.phrs.2022.106616. 36566926 10.1016/j.phrs.2022.106616

[24] Flegal KM, Carroll D, Kit BK, Ogden CL. Prevalence of obesity and trends in the distribution of body mass index among US adults, 1999–2010. JAMA. 2012;307:491. 10.1001/jama.2012.39. 22253363 10.1001/jama.2012.39

[25] Fulop GA, Kiss T, Tarantini S, Balasubramanian P, Yabluchanskiy A, Farkas E, Bari F, Ungvari Z, Csiszar A. Nrf2 deficiency in aged mice exacerbates cellular senescence promoting cerebrovascular inflammation. Geroscience. 2018;40:513. 10.1007/s11357-018-0047-6. 30470983 10.1007/s11357-018-0047-6 PMC6294722

[26] Galili O, Versari D, Sattler KJ, Olson ML, Mannheim D, McConnell JP, Chade AR, Lerman LO, Lerman A. Early experimental obesity is associated with coronary endothelial dysfunction and oxidative stress. Am J Physiol Heart Circ Physiol 2007: 292. 10.1152/ajpheart.00628.2006 10.1152/ajpheart.00628.2006 17012356

[27] Gill B, Harris A, Tredwin C, Gill P. Multimorbidity and oral health: need for new models of care. Fam Med Community Health. 2020: 8. 10.1136/fmch-2020-000387 10.1136/fmch-2020-000387 PMC7509975 32962988

[28] Giordano S, Vvictorzon M. Bariatric surgery in elderly patients: a systematic review. Clin Interv Aging. 2015: 10. 10.2147/CIA.S70313 10.2147/CIA.S70313 PMC4610711 26508845

[29] Glick M, Williams DM, Kleinman D V., Vujicic M, Watt RG, Weyant RJ. A new definition for oral health developed by the FDI World Dental Federation opens the door to a universal definition of oral health. Journal of the American Dental Association. 2016: 147. 10.1016/j.adaj.2016.10.001 10.1016/j.adaj.2016.10.001 27886668

[30] Grosso G, Laudisio D, Frias-Toral E, Barrea L, Muscogiuri G, Savastano S, Colao A. Anti-inflammatory nutrients and obesity-associated metabolic-inflammation: state of the art and future direction. Nutrients. 2022: 14. 10.3390/nu14061137 10.3390/nu14061137 PMC8954840 35334794

[31] Hales CM, Fryar CD, Carroll MD, Freedman DS, Ogden CL. Trends in obesity and severe obesity prevalence in US youth and adults by sex and age, 2007–2008 to 2015–2016. JAMA. 2018;319:1723–5. 29570750 10.1001/jama.2018.3060 PMC5876828

[32] Han DH, Lim SY, Sun BC, Paek DM, Kim HD. Visceral fat area-defined obesity and periodontitis among Koreans. J Clin Periodontol 2010: 37. 10.1111/j.1600-051X.2009.01515.x 10.1111/j.1600-051X.2009.01515.x 20041978

[33] Hasturk H, Kantarci A, Van Dyke TE. Oral inflammatory diseases and systemic inflammation: Role of the macrophage. Front Immunol. 2012: 3. 10.3389/fimmu.2012.00118 10.3389/fimmu.2012.00118 PMC3353263 22623923

[34] Haukka A, Heikkinen AM, Haukka J, Kaila M. Oral health indices predict individualised recall interval. Clin Exp Dent Res 2020: 6. 10.1002/cre2.319 10.1002/cre2.319 PMC7745075 32776480

[35] Head A, Fleming K, Kypridemos C, Schofield P, Pearson-Stuttard J, O’Flaherty M. Inequalities in incident and prevalent multimorbidity in England, 2004–19: a population-based, descriptive study. Lancet Healthy Longev 2021: 2. 10.1016/S2666-7568(21)00146-X 10.1016/S2666-7568(21)00146-X 36097998

[36] Hecker J, Freijer K, Hiligsmann M, Evers SMAA. Burden of disease study of overweight and obesity; the societal impact in terms of cost-of-illness and health-related quality of life. BMC Public Health 2022: 22. 10.1186/s12889-021-12449-2 10.1186/s12889-021-12449-2 PMC8740868 34996413

[37] Hilgert JB, Hugo FN, De Sousa MDLR, Bozzetti MC. Oral status and its association with obesity in Southern Brazilian older people. Gerodontology 2009: 26. 10.1111/j.1741-2358.2008.00226.x 10.1111/j.1741-2358.2008.00226.x 18371171

[38] Iwasaki M, Kimura Y, Ogawa H, Yamaga T, Ansai T, Wada T, Sakamoto R, Ishimoto Y, Fujisawa M, Okumiya K, Miyazaki H, Matsubayashi K. Periodontitis, periodontal inflammation, and mild cognitive impairment: a 5-year cohort study. J Periodontal Res 2019: 54. 10.1111/jre.12623 10.1111/jre.12623 30345659

[39] Jimenez M, Hu FB, Marino M, Li Y, Joshipura KJ. Prospective associations between measures of adiposity and periodontal disease. Obesity 2012: 20. 10.1038/oby.2011.291 10.1038/oby.2011.291 PMC3727227 21979390

[40] Jordan AR, Micheelis W. Fünfte deutsche mundgesundheitsstudie-(DMS IV). Deutscher Zahnärzte Verlag DÄV Köln 2016

[41] Jung CH, Lee MJ, Kang YM, Jang JE, Leem J, Hwang JY, Kim EH, Park JY, Kim HK, Lee WJ. The risk of incident type 2 diabetes in a Korean metabolically healthy obese population: the role of systemic inflammation. Journal of Clinical Endocrinology and Metabolism 2015: 100. 10.1210/jc.2014-3885 10.1210/jc.2014-3885 25490279

[42] Kassebaum NJ, Bernabé E, Dahiya M, Bhandari B, Murray CJL, Marcenes W. Global burden of untreated caries: a systematic review and metaregression. J Dent Res 2015: 94. 10.1177/0022034515573272 10.1177/0022034515573272 25740856

[43] Khongsirisombat N, Kiattavorncharoen S, Thanakun S. Increased oral dryness and negative oral health-related quality of life in older people with overweight or obesity. Dent J (Basel) 2022: 10. 10.3390/dj10120231 10.3390/dj10120231 PMC9776969 36547047

[44] Kim TN. Elderly obesity: is it harmful or beneficial? J Obes Metab Syndr. 2018: 27. 10.7570/JOMES.2018.27.2.84 10.7570/jomes.2018.27.2.84 PMC6489455 31089547

[45] Kitagawa M, Kurahashi T, Matsukubo T. Relationship between general health, lifestyle, oral health, and periodontal disease in adults: a large cross-sectional study in Japan. Bull Tokyo Dent Coll 2017: 58. 10.2209/tdcpublication.2016-2100 10.2209/tdcpublication.2016-2100 28381729

[46] Kossioni AE. The association of poor oral health parameters with malnutrition in older adults: a review considering the potential implications for cognitive impairment. Nutrients. 2018: 10. 10.3390/nu10111709 10.3390/nu10111709 PMC6266396 30413041

[47] Kreher D, Ernst BLV, Ziebolz D, Haak R, Ebert T, Schmalz G. Dental caries in adult patients with rheumatoid arthritis—a systematic review. J Clin Med. 2023: 12. 10.3390/jcm12124128 10.3390/jcm12124128 PMC10298950 37373822

[48] Kreher D, Ernst BLV, Ziebolz D, Haak R, de Fallois J, Ebert T, Schmalz G. Prevalence of dental caries in patients on renal replacement therapy—a systematic review. J Clin Med. 2023: 12. 10.3390/jcm12041507 10.3390/jcm12041507 PMC9967680 36836050

[49] Lee KS, Jha N, Kim YJ. Risk factor assessments of temporomandibular disorders via machine learning. Sci Rep 2021: 11. 10.1038/s41598-021-98837-5 10.1038/s41598-021-98837-5 PMC8492627 34611188

[50] Lee KS, Kim EK, Kim JW, Choi YH, Mechant AT, Song KB, Lee HK. The relationship between metabolic conditions and prevalence of periodontal disease in rural korean elderly. Arch Gerontol Geriatr 2014: 58. 10.1016/j.archger.2013.08.011 10.1016/j.archger.2013.08.011 24075494

[51] Linden G, Patterson C, Evans A, Kee F. Obesity and periodontitis in 60–70-year-old men. J Clin Periodontol 2007: 34. 10.1111/j.1600-051X.2007.01075.x 10.1111/j.1600-051X.2007.01075.x 17403015

[52] Liu F, Song S, Ye X, Huang S, He J, Wang G, Hu X. Oral health-related multiple outcomes of holistic health in elderly individuals: an umbrella review of systematic reviews and meta-analyses. Front Public Health. 2022: 10. 10.3389/fpubh.2022.1021104 10.3389/fpubh.2022.1021104 PMC9650948 36388333

[53] Magnusson KR, Hauck L, Jeffrey BM, Elias V, Humphrey A, Nath R, Perrone A, Bermudez LE. Relationships between diet-related changes in the gut microbiome and cognitive flexibility. Neuroscience 2015: 300. 10.1016/j.neuroscience.2015.05.016 10.1016/j.neuroscience.2015.05.016 25982560

[54] Minagawa K, Iwasaki M, Ogawa H, Yoshihara A, Miyazaki H. Relationship between metabolic syndrome and periodontitis in 80-year-old Japanese subjects. J Periodontal Res 2015: 50. 10.1111/jre.12190 10.1111/jre.12190 24815529

[55] Misra A, Khurana L. Obesity-related non-communicable diseases: South Asians vs White Caucasians. Int J Obes. 2011: 35. 10.1038/ijo.2010.135 10.1038/ijo.2010.135 20644557

[56] Müller F, Naharro M, Carlsson GE. What are the prevalence and incidence of tooth loss in the adult and elderly population in Europe? Clin Oral Implants Res. 2007: 18. 10.1111/j.1600-0501.2007.01459.x 10.1111/j.1600-0501.2007.01459.x 17594365

[57] Muñoz-Torres FJ, Jiménez MC, Rivas-Tumanyan S, Joshipura KJ. Associations between measures of central adiposity and periodontitis among older adults. Community Dent Oral Epidemiol 2014: 42. 10.1111/cdoe.12069 10.1111/cdoe.12069 PMC3949210 24010953

[58] Nair GR, Jadhav SL, Palal D, Rathod H, Verma P, Bhawalkar J, Rathi MA, Ray S, Madamanchi D. The role of neck circumference as a screening tool for obesity in female adults: a cross-sectional study in Western Maharashtra. Cureus. 2024;16: e65814. 39219905 10.7759/cureus.65814 PMC11362821

[59] Oikarinen R, Syrjälä AM, Komulainen K, Knuuttila M, Ruoppi P, Hartikainen S, Sulkava R, Ylöstalo P. Body mass index and periodontal infection in a sample of non-smoking older individuals. Oral Dis 2014: 20. 10.1111/odi.12108 10.1111/odi.12108 23577782

[60] Okifuji A, Hare BD. The association between chronic pain and obesity. J Pain Res. 2015: 8. 10.2147/JPR.S55598 10.2147/JPR.S55598 PMC4508090 26203274

[61] Otten RVRDSL. Amsterdam efficient deduplication (AED) method (version 1). Zenodo. 2019.

[62] Ouzzani M, Hammady H, Fedorowicz Z, Elmagarmid A. Rayyan-a web and mobile app for systematic reviews. Syst Rev 2016: 5. 10.1186/s13643-016-0384-4 10.1186/s13643-016-0384-4 PMC5139140 27919275

[63] Page MJ, McKenzie JE, Bossuyt PM, Boutron I, Hoffmann TC, Mulrow CD, Shamseer L, Tetzlaff JM, Akl EA, Brennan SE, Chou R, Glanville J, Grimshaw JM, Hróbjartsson A, Lalu MM, Li T, Loder EW, Mayo-Wilson E, McDonald S, McGuinness LA, Stewart LA, Thomas J, Tricco AC, Welch VA, Whiting P, Moher D. The PRISMA 2020 statement: an updated guideline for reporting systematic reviews. The BMJ. 2021: 372. 10.1136/bmj.n71 10.1136/bmj.n71 PMC8005924 33782057

[64] Palanisamy S. The impact of estrogen on periodontal tissue integrity and inflammation—a mini review. Frontiers in Dental Medicine. 2025;6:1455755. 40045936 10.3389/fdmed.2025.1455755 PMC11880030

[65] Partridge L, Deelen J, Slagboom PE. Facing up to the global challenges of ageing. Nature. 2018: 561. 10.1038/s41586-018-0457-8 10.1038/s41586-018-0457-8 30185958

[66] Peruchi CTR, Poli-Frederico RC, Cardelli AAM, Fracasso MDLC, Bispo CGC, Neves-Souza RD, Cardoso JR, Maciel SM. Association between oral health status and central obesity among Brazilian independent-living elderly. Braz Oral Res 2016: 30. 10.1590/1807-3107BOR-2016.VOL30.0116 10.1590/1807-3107BOR-2016.vol30.0116 27783768

[67] Petersen PE, The World Oral Health Report. Continuous improvement of oral health in the 21st century - the approach of the WHO Global Oral Health Programme. Community Dent Oral Epidemiol. 2003;2003:31. 10.1046/j.2003.com122.x. 10.1046/j..2003.com122.x 15015736

[68] Polzer I, Schwahn C, Völzke H, Mundt T, Biffar R. The association of tooth loss with all-cause and circulatory mortality. Is there a benefit of replaced teeth? A systematic review and meta-analysis. Clin Oral Investig. 2012: 16. 10.1007/s00784-011-0625-9 10.1007/s00784-011-0625-9 22086361

[69] Rivas-Tumanyan S, Campos M, Zevallos JC, Joshipura KJ. Periodontal disease, hypertension, and blood pressure among older adults in Puerto Rico. J Periodontol 2013: 84. 10.1902/jop.2012.110748 10.1902/jop.2012.110748 PMC3561508 22548584

[70] Rodrigues Junior HL, Scelza MFZ, Boaventura GT, Custódio SM, Moreira EAM, de Lima Oliveira D. Relation between oral health and nutritional condition in the elderly. Journal of Applied Oral Science 2012: 20. 10.1590/S1678-77572012000100008 10.1590/S1678-77572012000100008 PMC3928770 22437676

[71] Rondanelli M. Review on microbiota and effectiveness of probiotics use in older. World J Clin Cases 2015: 3. 10.12998/wjcc.v3.i2.156 10.12998/wjcc.v3.i2.156 PMC4317609 25685762

[72] Sakib MN, Shooshtari S, St John P, Menec V. The prevalence of multimorbidity and associations with lifestyle factors among middle-aged Canadians: an analysis of Canadian longitudinal study on aging data. BMC Public Health 2019: 19. 10.1186/s12889-019-6567-x 10.1186/s12889-019-6567-x PMC6394050 30819126

[73] Salas MMS, Vargas-Ferreira F, Nascimento GG, Huysmanns MC, Demarco FF. Tooth erosion association with obesity: findings from a Brazilian survey in schoolchildren. Pesqui Bras Odontopediatria Clin Integr 2018: 18. 10.4034/PBOCI.2018.181.25

[74] Sato M, Iwasaki M, Yoshihara A, Miyazaki H. Association between periodontitis and medical expenditure in older adults: a 33-month follow-up study. Geriatr Gerontol Int 2016: 16. 10.1111/ggi.12569 10.1111/ggi.12569 26272677

[75] Schmalz G, Li S, Ziebolz D. Oral health-related quality of life in patients after stroke— a systematic review. J Clin Med. 2022: 11. 10.3390/jcm11051415 10.3390/jcm11051415 PMC8911029 35268507

[76] Schneider C, Zemp E, Zitzmann NU. Oral health improvements in Switzerland over 20 years. Eur J Oral Sci 2017: 125. 10.1111/eos.12327 10.1111/eos.12327 28045197

[77] Sheng X, Xiao X, Song X, Qiao L, Zhang X, Zhong H. Correlation between oral health and quality of life among the elderly in Southwest China from 2013 to 2015. Medicine (United States) 2018: 97. 10.1097/MD.0000000000010777 10.1097/MD.0000000000010777 PMC6392902 29794757

[78] Singh H, Torralba MG, Moncera KJ, DiLello L, Petrini J, Nelson KE, Pieper R. Gastro-intestinal and oral microbiome signatures associated with healthy aging. Geroscience 2019: 41. 10.1007/s11357-019-00098-8 10.1007/s11357-019-00098-8 PMC6925087 31620923

[79] Smith SM, Sumar B, Dixon KA. Musculoskeletal pain in overweight and obese children. Int J Obes. 2014: 38. 10.1038/ijo.2013.187 10.1038/ijo.2013.187 PMC3884137 24077005

[80] Starr ME, Evers BM, Saito H. Age-associated increase in cytokine production during systemic inflammation: adipose tissue as a major source of IL-6. Journals of Gerontology - Series A Biological Sciences and Medical Sciences 2009: 64. 10.1093/gerona/glp046 10.1093/gerona/glp046 PMC2844135 19377014

[81] StataCorp L. Mata reference manual. College Station, TX, StataCorp LLC 2017

[82] Stout MB, Justice JN, Nicklas BJ, Kirkland JL. Physiological aging: links among adipose tissue dysfunction, diabetes, and frailty. Physiology. 2017: 32. 10.1152/physiol.00012.2016 10.1152/physiol.00012.2016 PMC5338596 27927801

[83] Sun C, Kovacs P, Guiu-Jurado E. Genetics of body fat distribution: comparative analyses in populations with European, Asian and African ancestries. Genes (Basel). 2021: 12. 10.3390/genes12060841 10.3390/genes12060841 PMC8228180 34072523

[84] Sung J, Ho CT, Wang Y. Preventive mechanism of bioactive dietary foods on obesity-related inflammation and diseases. Food Funct 2018: 9. 10.1039/c8fo01561a 10.1039/c8fo01561a 30403220

[85] Surmi BK, Hasty AH. Macrophage infiltration into adipose tissue: initiation, propagation and remodeling. Future Lipidol. 2008: 3. 10.2217/17460875.3.5.545 10.2217/17460875.3.5.545 PMC2575346 18978945

[86] Tarantini S, Tucsek Z, Valcarcel-Ares MN, Toth P, Gautam T, Giles CB, Ballabh P, Wei JY, Wren JD, Ashpole NM, Sonntag WE, Ungvari Z, Csiszar A. Circulating IGF-1 deficiency exacerbates hypertension-induced microvascular rarefaction in the mouse hippocampus and retrosplenial cortex: implications for cerebromicrovascular and brain aging. Age (Omaha) 2016: 38. 10.1007/s11357-016-9931-0 10.1007/s11357-016-9931-0 PMC5061685 27613724

[87] Tarantini S, Valcarcel-Ares MN, Yabluchanskiy A, Tucsek Z, Hertelendy P, Kiss T, Gautam T, Zhang XA, Sonntag WE, De Cabo R, Farkas E, Elliott MH, Kinter MT, Deak F, Ungvari Z, Csiszar A. Nrf2 deficiency exacerbates obesity-induced oxidative stress, neurovascular dysfunction, blood-brain barrier disruption, neuroinflammation, amyloidogenic gene expression, and cognitive decline in mice, mimicking the aging phenotype. Journals of Gerontology - Series A Biological Sciences and Medical Sciences 2018: 73. 10.1093/gerona/glx177 10.1093/gerona/glx177 PMC6001893 29905772

[88] Teixeira FCF, Marin-Leon L, Gomes EP, Pedrão AMN, Pereira A da C, Francisco PMSB. Relationship between periodontitis and subclinical risk indicators for chronic non-communicable diseases. Braz Oral Res 2020: 34. 10.1590/1807-3107BOR-2020.VOL34.0058 10.1590/1807-3107bor-2020.vol34.0058 32578801

[89] Ticinesi A, Tana C, Nouvenne A, Prati B, Lauretani F, Meschi T. Gut microbiota, cognitive frailty and dementia in older individuals: a systematic review. Clin Interv Aging. 2018: 13. 10.2147/CIA.S139163 10.2147/CIA.S139163 PMC6120508 30214170

[90] Tsai KZ, Su FY, Cheng WC, Lin YP, Lin GM. Association of hepatic and systemic inflammation with localized stage II/III periodontitis in young males: the CHIEF oral health study. J Clin Periodontol 2022: 49. 10.1111/jcpe.13556 10.1111/jcpe.13556 34611936

[91] Tucsek Z, Toth P, Tarantini S, Sosnowska D, Gautam T, Warrington JP, Giles CB, Wren JD, Koller A, Ballabh P, Sonntag WE, Ungvari Z, Csiszar A. Aging exacerbates obesity-induced cerebromicrovascular rarefaction, neurovascular uncoupling, and cognitive decline in mice. Journals of Gerontology - Series A Biological Sciences and Medical Sciences 2014: 69. 10.1093/gerona/glu080 10.1093/gerona/glu080 PMC4204615 24895269

[92] United Nations DoEaSA PD. World Population Ageing, 2019. Highlights (ST/ESA/SER.A/430). New York 2019

[93] Valcarcel-Ares MN, Tucsek Z, Kiss T, Giles CB, Tarantini S, Yabluchanskiy A, Balasubramanian P, Gautam T, Galvan V, Ballabh P, Richardson A, Freeman WM, Wren JD, Deak F, Ungvari Z, Csiszar A. Obesity in aging exacerbates neuroinflammation, dysregulating synaptic function-related genes and altering eicosanoid synthesis in the mouse hippocampus: potential role in impaired synaptic plasticity and cognitive decline. Journals of Gerontology - Series A Biological Sciences and Medical Sciences 2019: 74. 10.1093/gerona/gly127 10.1093/gerona/gly127 PMC6376091 29893815

[94] Vieira AT, Castelo PM, Ribeiro DA, Ferreira CM. Influence of oral and gut microbiota in the health of menopausal women. Front Microbiol. 2017: 8. 10.3389/fmicb.2017.01884 10.3389/fmicb.2017.01884 PMC5625026 29033921

[95] Walrand S, Guillet C, Salles J, Cano N, Boirie Y. Physiopathological mechanism of sarcopenia. Clin Geriatr Med. 2011: 27. 10.1016/j.cger.2011.03.005 10.1016/j.cger.2011.03.005 21824553

[96] Wang X, Cheng Z. Cross-sectional studies: strengths, weaknesses, and recommendations. Chest. 2020: 158. 10.1016/j.chest.2020.03.012 10.1016/j.chest.2020.03.012 32658654

[97] Wang Y, Beydoun MA. The obesity epidemic in the United States - gender, age, socioeconomic, racial/ethnic, and geographic characteristics: a systematic review and meta-regression analysis. Epidemiol Rev. 2007: 29. 10.1093/epirev/mxm007 10.1093/epirev/mxm007 17510091

[98] Wang YC, Colditz GA, Kuntz KM. Forecasting the obesity epidemic in the aging U.S. population. Obesity 2007: 15. 10.1038/oby.2007.339 10.1038/oby.2007.339 18070778

[99] Yamazaki Y, Baker DJ, Tachibana M, Liu CC, Van Deursen JM, Brott TG, Bu G, Kanekiyo T. Vascular cell senescence contributes to blood-brain barrier breakdown. Stroke 2016: 47. 10.1161/STROKEAHA.115.010835 10.1161/STROKEAHA.115.010835 PMC4811685 26883501

[100] Yang Q, Wang X, Li C, Wang X. A cross-sectional study on the relationship between visceral adiposity index and periodontitis in different age groups. Sci Rep 2023: 13. 10.1038/s41598-023-33082-6 10.1038/s41598-023-33082-6 PMC10086006 37037870

[101] Zamboni M, Mazzali G, Fantin F, Rossi A, Di Francesco V. Sarcopenic obesity: a new category of obesity in the elderly. Nutrition, Metabolism and Cardiovascular Diseases. 2008: 18. 10.1016/j.numecd.2007.10.002 10.1016/j.numecd.2007.10.002 18395429

[102] Obesity: preventing and managing the global epidemic. Report of a WHO consultation. World Health Organ Tech Rep Ser 2000: 894. 11234459

